# Crowdsourced Comparison of Aesthetic Outcomes of Traditional Transverse Versus Skin-Reducing Mastectomy Incision Patterns Following Implant-Based Breast Reconstruction

**DOI:** 10.7759/cureus.79924

**Published:** 2025-03-02

**Authors:** Blake T Dunson, Daniel P Zaki, Mario S Blondin, Mary L Duet, Thomas Steele, Christine V Pestana, Ivo A Pestana

**Affiliations:** 1 Plastic and Reconstructive Surgery, Atrium Health Wake Forest Baptist, Winston-Salem, USA; 2 Breast Surgery, Lynn Cancer Institute, Boca Raton, USA; 3 Plastic and Reconstructive Surgery, Lynn Cancer Institute, Boca Raton, USA

**Keywords:** crowdsource, implant-based breast reconstruction, mastectomy scar, plastic surgery crowdsource, skin-reducing mastectomy, skin-reducing mastectomy incision patterns, transverse mastectomy incision patterns, vertical mastectomy incision-patterns

## Abstract

Introduction

Advancements in reconstructive breast surgery have made postoperative cosmesis and patient satisfaction critical outcome measures. Skin-sparing mastectomy (SSM) incision patterns may be classified into the traditional transverse incision, or skin-reducing patterns. The aim of this study is to identify preferential trends among the public regarding aesthetic outcomes between incision patterns following implant-based breast reconstruction (IBBR).

Methods

Twelve patients who underwent IBBR following SSM were included, six with a transverse incision pattern, and six with skin-reducing mastectomy (SRM) patterns. Patients were matched for age, body mass index (BMI), American Society of Anesthesiology (ASA) physical status classification system, comorbidities, Regnault ptosis grade, and chemotherapy/radiation status. A survey was created via the Research Electronic Data Capture (RedCap) database to assess outcomes in seven categories: symmetry, volume, projection, shape, skin quality, scar pattern, and overall aesthetic rating. The survey was distributed via social media and the Amazon MTurk crowdsourcing platform.

Results

The survey collected 1,194 responses, predominantly from females under 40 years of age, with a balanced distribution of respondents with and without healthcare experience. Across all assessed categories, SRM patterns were rated more favorably than transverse incisions (p < 0.001). Notably, SRM patterns were preferred in scenarios involving nipple-areolar complex (NAC) reconstruction.

Conclusions

The SRM was found to be more aesthetically pleasing to the general public regardless of age, gender, or healthcare experience. These results should be considered when planning incision patterns for patients undergoing mastectomy.

## Introduction

Skin-preserving procedures have emerged as a pivotal advancement in the surgical treatment of breast cancer, marking a paradigm shift from traditional mastectomy approaches [1,2]. As the understanding of breast cancer progressed, Halsted’s radical mastectomy gradually evolved into less extensive surgical interventions. Today, most patients undergo skin-sparing mastectomy (SSM) or nipple-sparing mastectomy (NSM), which are the latest evolutions in mastectomy techniques [2]. This shift is driven by the improved cosmetic appearance associated with the preservation of the breast skin, without compromising oncologic outcomes [2,3]. Both SSM and NSM have shown similar outcomes to non-conservative mastectomies in terms of recurrence rates, underscoring their efficacy and safety [4]. NSM is the pinnacle of skin preservation and is associated with excellent patient-reported outcomes [5-7]. However, NSM is not always a viable option. The SSM remains the most commonly performed mastectomy technique performed today.

In the context of SSM, various incision patterns have been explored to optimize reconstructive outcomes, including the traditional transverse elliptical incision and skin-reducing patterns. The transverse incision is centered around the nipple-areolar complex (NAC), whereas skin-reducing mastectomy (SRM) patterns employ vertical and/or transverse incisions along the inframammary fold, similar to those utilized in breast reduction and mastopexy procedures [8-10]. Despite their technical and oncological safety, SRM incision patterns are less frequently employed compared to the traditional transverse elliptical incision [9,10]. The choice of incision pattern is critical, as it directly impacts postoperative cosmesis, a key outcome measure in reconstructive breast surgery [11-13].

Crowdsourcing has emerged as an invaluable tool in plastic and reconstructive surgery, enabling the collection of diverse opinions on a large scale, which proves particularly beneficial in assessing aesthetic outcomes of surgical procedures. We aim to leverage crowdsourcing to identify if there is a preference among the public regarding aesthetic outcomes between the traditional transverse and skin-reducing incision patterns following implant-based breast reconstruction (IBBR). Our hypothesis is that SRM incision patterns will be identified as more aesthetically pleasing to the general public.

This article was originally presented as an abstract at the American Society of Breast Surgeons meeting in Boston, MA, USA, from April 26 to April 30, 2023.

## Materials and methods

Dataset

A retrospective review of the electronic medical record, approved by the Institutional Review Board (IRB00073220), was conducted at a single institution (Atrium Health Wake Forest Baptist, Winston-Salem, USA). Patients who underwent IBBR following SSM between 2012 and 2021 were identified through an audit list utilizing International Classification of Diseases (ICD)-9/10 and Current Procedural Terminology (CPT) codes. Patients between 18 and 99 years of age who had undergone mastectomy followed by bilateral IBBR were included. Exclusion criteria included patients under 18 and over 99 years old, who had not undergone breast reconstruction surgery within the specified time frame. No patients included had a latissimus dorsi flap used in their reconstruction. Relevant data and postoperative photos were extracted and collected from the electronic medical record and managed using the Research Electronic Data Capture (REDCap) database. All patients included consented to participation in the study.

Survey design

**Figure 1:**
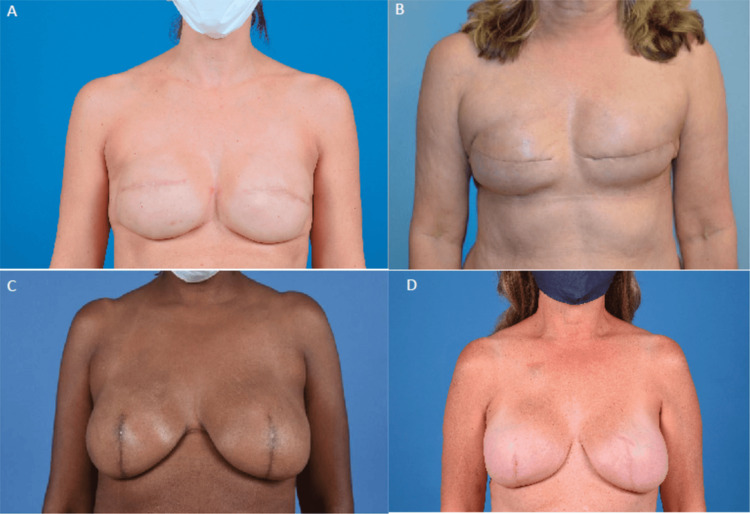
Sample survey comparison of traditional transverse elliptical incision pattern result (A, B) and skin-reducing incision pattern result (C, D) Age of patients: A = 36 years old, B = 60 years old, C = 50 years old, D = 55 years old.

A series of patients with a transverse elliptical incision pattern and SRM patterns were included. The patients included were those of multiple oncologic breast surgeons and plastic surgeons. Patients were matched for age, body mass index (BMI), preoperative breast cup/ptosis grade, comorbidities, American Society of Anesthesia (ASA), and chemotherapy/radiation status. A survey of de-identified postoperative patient photos was created via REDCap to assess aesthetic outcomes (Figure 1). The Validated Breast Aesthetic Scale was utilized to create survey response choices [14]. All images were taken in a professional photo suite, using a standardized frontal view and two lateral views as part of surgical follow-up (three photos per patient included). Images were cropped in a uniform fashion to standardize position, lighting, and form.

Crowdsourcing

The survey was distributed via social media and the Amazon MTurk (www.mturk.com) crowdsourcing platform. Respondents were aged at least 18 years and were categorized based on the presence or absence of healthcare work experience, which was ascertained by a series of three questions. First, respondents indicated if they presently work or have studied in healthcare. Subsequently, they chose a professional category aligning with their healthcare role: Physician, Nurse, Medical Support Staff, Student (medical, physician assistant, certified registered nurse anesthetist, nurse practitioner, nursing), or Other. Those selecting "Other" were prompted to provide a free-text response to specify their healthcare field of experience. Respondents' age and gender were also collected. All respondents consented to participate in the survey.

Assessment of aesthetic outcome

Respondents rated each set of photos on a numerical scale, with descriptions corresponding to each discrete score (Table 1). Six breast-related categories were assessed in this manner: breast symmetry, volume, projection, shape, skin quality, and scar pattern. Overall aesthetic rating was conducted using a visual analog scale ranging from 1 to 10 (with 1 indicating the most unacceptable and 10 indicating exceptional). Additionally, each respondent rated which scar patterns, overall appearance, they preferred when shown two patients with reconstructed NACs.

**Table 1:** Validated breast aesthetic scale descriptions with corresponding scores

	1	2	3	4
Symmetry of overall breasts	Severely asymmetric	Moderately asymmetric	Slightly asymmetric	Symmetric
Symmetry of breast volume	Severely asymmetric	Moderately asymmetric	Slightly asymmetric	Symmetric
Projection	Insufficient projection	Excessive projection	Projection proportional to body habitus	N/A
Shape	Insufficient cone shape	Excessive cone shape	Satisfactory cone shape	N/A
Skin quality	Poor skin quality	Satisfactory skin quality	Excellent skin quality	N/A
Scar pattern	Predominately vertical	Combination of vertical and transverse	Predominantly transverse	N/A

Data analyses

Once survey responses were recorded and analyzed, the sample was stratified by the incision pattern employed, and respondent aesthetic evaluation was calculated for each of the selected covariates using logistic regression. Student t-tests were used for aesthetic comparison of mastectomy incision patterns. For patient demographics, a combination of Chi-square and analysis of variance (ANOVA) tests was used, depending on whether the dataset was categorical or numerical. A p-value of less than 0.05 was considered statistically significant.

## Results

Demographics

A total of 12 patients were included, six with a transverse elliptical incision pattern and six with SRM patterns. Of these 12 patients, 10 were White or Caucasian individuals, and two were Black or African American individuals. Average age, follow-up, and BMI were 45.57 years, 26.78 months, and 24.01 in the transverse incision group, respectively, and 44.43 years, 11.21 months, and 25.7 in the SRM incision pattern group, respectively (p > 0.05 for all). There were no differences in comorbidities between the two groups (Table 2).

**Table 2:** Breast reconstruction patient characteristics A combination of Chi-square and ANOVA tests were used, depending on whether the dataset was categorical or numerical. Abbreviations: ASA, American Society of Anesthesiologists; BMI, Body Mass Index; D/O, Disorder; DVT, Deep Venous Thrombosis; PE, Pulmonary Embolism; ANOVA, Analysis of Variance

Characteristic	Transverse	SRM	p-value
Total patients, n	6	6	NS
Age, mean (SD), y	45.57 ± 11.52	44.43 ± 8.36	0.8353
Follow-up, mean ± SD (months)	26.78 ± 21.17	11.21 ± 8.16	0.0944
BMI, mean ± SD, kg/m^2^	24.01 ± 2.52	25.7 ± 2.78	0.2511
ASA status, mean ± SD	2.14 ± 0.38	2.14 ± 0.38	NS
Neoadjuvant chemotherapy, n (%)	1 (16.7%)	2 (33.3%)	0.5517
Adjuvant chemotherapy, n (%)	1 (16.7%)	0 (0%)	0.337
Radiotherapy, n (%)	0 (0%)	0 (0%)	NS
Diabetes, n (%)	0 (0%)	0 (0%)	NS
History of smoking, n (%)	3 (50%)	0 (0%)	0.0554
Active tobacco use, n (%)	0 (0%)	0 (0%)	NS
History of DVT/PE, n (%)	0 (0%)	1 (16.7%)	0.337
Hypertension, n (%)	0 (0%)	0 (0%)	NS
Autoimmune D/O, n (%)	1 (16.7%)	1 (16.7%)	NS
Hyperlipidemia, n (%)	0 (0%)	0 (0%)	NS
Mental health D/O, n (%)	3 (50%)	2 (33.3%)	0.611

Respondents

A total of 1,194 survey responses were recorded and analyzed. Respondents tended to be female, less than 40 years of age, and similarly distributed in terms of healthcare experience (Table 3). Respondents, with or without healthcare experience, could accurately identify the difference between scar patterns.

**Table 3:** Respondent demographics

Respondent demographics	Respondents (n)
All	1,194
Age (years)	
18-39	844
40-59	304
60+	46
Gender	
Female	734
Male	444
Non-binary	14
Other	2
Prior healthcare experience	
Yes	659
No	535

Aesthetic outcomes

Respondents rated the SRM higher compared to the transverse pattern in every breast-related category: symmetry, volume, projection, shape, skin quality, scar pattern, and overall aesthetic rating (Table 4). When comparing aesthetic preference of results between incision patterns with the NAC reconstructed, skin-reducing incision patterns were preferred.

**Table 4:** Aesthetic comparison of transverse vs. SRM incision patterns Scores for breast symmetry ranged from 1 to 4. Scores for breast volume, projection, shape, and skin quality ranged from 1 to 3. t-tests were used to compare breast characteristics between the transverse and SRM incision patterns. The remaining 316 respondents rated the two-incision patterns as equally pleasing when shown two patients with reconstructed NACs. Abbreviations: NAC, Nipple-Areolar Complex; SRM, Skin-Reducing Mastectomy

	Transverse	SRM	p-value
Breast volume symmetry	2.50	2.91	<0.001
Overall breast symmetry	2.48	2.82	<0.001
Breast projection	1.88	2.46	<0.001
Breast cone shape	1.73	2.38	<0.001
Skin quality	1.86	2.16	<0.001
Correct identification of scar pattern, n (%)	795 (66.6)	1,030 (86.3)	<0.001
Overall rating (1-10)	5.56	6.89	<0.001
Preference with NAC in place, n (%)	350 (29.3)	528 (44.2)	<0.001

## Discussion

High patient satisfaction is the ultimate goal for reconstructive breast surgeons, which is directly influenced by the type of mastectomy performed. Although NSM has been shown to achieve the highest patient satisfaction, it is not always a viable option. Therefore, selecting the appropriate incision pattern for SSM is crucial, as it impacts scar burden and location, cosmesis, and overall reconstructive results. Regarding patient satisfaction, limited studies utilizing the BREAST-Q survey have shown no difference in patient-reported outcomes when comparing vertical to non-vertical incision patterns [15,16]. However, patient satisfaction is influenced by various factors, and the BREAST-Q survey may not be ideal for evaluating this aspect, as it is not specifically designed to assess the visibility of mastectomy scars.

The transverse mastectomy incision pattern is often the default choice [11,15,16]. The safety profile of transverse versus vertical incision patterns has been shown to not be significantly different [15,16]. Dayicioglu et al. conducted a retrospective review of 167 patients who underwent vertical (n = 38) vs. non-vertical (n = 129) SSM incision patterns with tissue expander reconstruction. They found no difference in complication rates and mastectomy skin necrosis. Additionally, vertical incisions did not interfere with achieving adequate tumor margins, and there were no differences in breast weight or implant volume between the cohorts [15].

Despite the transverse incision pattern being more commonly utilized, SRM incisions offer several advantages in breast reconstruction. The scars in SRM incisions provide hidden scars with minimal impact on aesthetics [13]. Literature based on the opinions of surgeons and surgical trainees has shown SRM incision patterns to be considered aesthetically superior to transverse incision patterns [13,17]. Lotan et al. conducted a study to determine the optimal mastectomy incision design for aesthetic outcomes [13]. Twenty plastic surgeons evaluated the aesthetic results, considering scar visibility and position, using before-and-after photos of reconstructed breasts with nine different commonly utilized incision patterns. The study found that hidden scars and vertical scars were preferred over transverse scars and suggested the use of transverse incisions only when a preexisting scar is present [13]. Bourne et al. conducted a retrospective review of 77 patients who underwent immediate autologous breast reconstruction, either using a vertical or transverse “racquet” incision pattern after SSM [17]. Twenty-three plastic surgery trainees evaluated aesthetic outcomes based on a Likert scale of scar appearance, shape, preoperative versus postoperative aesthetic comparison, and overall aesthetic outcome. Vertical incisions demonstrated significantly better scar scores and similar shape and overall postoperative aesthetic appearance despite greater ptosis preoperatively in the vertical group [17]. Notably, the transverse incision can be modified to an oblique incision to provide a better aesthetic result [11]. Additionally, vertical incisions facilitate future corrective procedures for recurrent ptosis and offer adequate access to various surgical interventions. Conversely, transverse incisions are associated with lower aesthetic quality and may result in scar contraction, potentially leading to nipple malposition and limiting options for ptosis correction [13]. There remains a notable gap in the literature regarding comprehensive evaluations of cosmetic outcomes from the patient's and public's perspective, as no study to date has compared the cosmetic outcomes of SRM following IBBR [18].

Our study leverages crowdsourcing to bridge this gap, providing valuable insights into public and patient aesthetic preferences for SRM vs. traditional transverse incisions. While the surgeon's viewpoint is vital in assessing aesthetic breast results, the views of the general public hold equal, if not greater, importance and may not always coincide with those of the surgeon [19-22]. By sampling responses from over 1,000 respondents, we were able to quickly identify if there existed a preference among the public regarding aesthetic outcomes between the traditional transverse and skin-reducing incision patterns following IBBR.

Our findings suggest that the general public deemed SRM incision patterns as more aesthetically pleasing, irrespective of respondent age, gender, or healthcare experience. Significant differences favoring SRM were observed across all aesthetic breast rating categories, even when NAC reconstruction was performed. These findings are congruent with the literature, which has indicated that surgeons and the public prefer SRM patterns over transverse incision patterns in breast reconstruction [12,13,16,17]. In a recent study, Lakatta et al. conducted a crowdsourced evaluation comparing vertical and transverse scar patterns in individuals who had undergone autologous breast reconstruction [16]. In their survey of 982 participants, the researchers discovered a consistent preference for vertical incisions over transverse ones, irrespective of respondent demographics. They also noted higher BREAST-Q scores among individuals in the vertical incision group. Additionally, there were no significant differences in postoperative breast complications between patients with horizontal or vertical incisions when considering wound, infection, seroma, hematoma, fat necrosis, or overall complications [16]. Our study confirms the findings of Lakatta et al. and adds to theirs, as, to the best of our knowledge, no other study has compared SRM and transverse incision patterns in patients who have undergone IBBR. Potential explanations for these findings could be attributed to variations in breast morphology and the visibility of scars. While utilizing a transverse incision pattern may result in a perceived flattened appearance of the breast, we assert the superior outcomes associated with SRM patterns stem from diminished visibility, possible enhanced concealment under clothing (depending on the height and location of vertical incisions), and these patterns tend to result in coning of the breast, which is more consistent with a natural breast appearance [11,12]. Further, patients might associate a vertical incision with the characteristics of a mastopexy or breast reduction, while a transverse incision may carry the stigma of a mastectomy.

Crowdsourcing has recently emerged as a reliable and powerful tool for accumulating and analyzing aesthetic outcomes in breast reconstruction [23-27]. Moreover, it is rapid, avoids expert bias [23,28,29], and can reconcile insight regarding what constitutes a “desirable” aesthetic outcome between the varying perspectives of the surgeon, patient, and general public. Unsurprisingly, this methodology has proven especially valuable in evaluating outcomes of cosmetic and reconstructive procedures, where subjective aesthetic judgments play a crucial role in determining success [1]. A systematic review by Villavisanis et al. revealed that one-third, or 15 out of 45 studies, of crowdsourcing research in plastic surgery is dedicated to aesthetic or breast surgery [30]. The effectiveness and efficiency of this methodology are illustrated by Teotia et al., who collected 901 layperson evaluations in just over a day, whereas acquiring expert assessments required more than 13 months [23]. This notable contrast emphasizes the substantial potential of crowdsourcing to expedite and enhance research capabilities in reconstructive breast surgery. In fact, this allows for the potential guidance of clinical practice towards techniques aligned with patient and public aesthetic ideals, which is a feat that seems insurmountable by conventional research methods. Other categories that have been explored via crowdsourcing include functional outcomes, public perceptions on numerous topics, and patient educational materials [30].

We recognize several limitations in our study. First, despite the substantial potential of crowdsourcing, it comes with inherent limitations. The diverse pool of contributors with varying expertise and motivations may introduce discrepancies and biases. Additionally, respondents might lack expertise, resulting in inaccurate or less-informed responses. Nevertheless, the strength of crowdsourcing lies in the large volume of raters, minimizing the impact of individual biases and confounding factors to a statistically insignificant level [23]. Importantly, the ability to discern differences between scar patterns did not vary significantly between respondents with healthcare experience and those without, indicating a general aptitude for recognizing aesthetic distinctions among the surveyed population. Second, the absence of data on the racial/ethnic demographics of respondents in our study introduces a potential source of selection bias. Future data collection efforts should prioritize gathering this information, as it holds the potential to enhance physicians' understanding of the diverse populations they serve. Taken together, this may ultimately lead to improved outcomes for their patients. Further, we did not gather data on whether respondents were acquainted with individuals who had undergone breast reconstruction, a factor known to positively influence aesthetic ratings [16]. Third, the postoperative images featured in the study were curated from various surgeons, potentially influencing individual aesthetic outcomes due to variations in surgeon approach to mastectomy and IBBR. However, this study ensured that all patients included were matched according to age, BMI, preoperative breast cup/ptosis grade, comorbidities, ASA, and chemotherapy/radiation status. This matching process minimized individual aesthetic variations among patients, enabling a focused examination of the impact of the incision pattern. Despite these limitations, this study offers evidence that the general public perceives SRM incision patterns as more aesthetically pleasing. This suggests that, when feasible for the patient, employing SRM incision patterns may be beneficial, given the absence of differences in complication rates, tumor margins, and its broad applicability [15].

## Conclusions

Our study constitutes a comprehensive crowdsourced evaluation of aesthetic outcomes following SSM with either traditional transverse or SRM incision patterns in the context of IBBR. The results unequivocally favor SRM patterns as more aesthetically pleasing, transcending variations in age, gender, or healthcare background of the respondents. This study suggests that, where clinically appropriate, the adoption of SRM incision patterns could enhance patient satisfaction with the aesthetic results of breast reconstruction.
